# Role of S-methylisothiourea (SMT) in renal ischemia/reperfusion injury in rats

**DOI:** 10.15171/jrip.2016.07

**Published:** 2016-02-28

**Authors:** Fatemeh Kanani, Faezeh Fazelnia, Mohaddeseh Mojarradfard, Mehdi Nematbakhsh, Fatemeh Moslemi, Fatemeh Eshraghi-Jazi, Ardeshir Talebi

**Affiliations:** ^1^Water & Electrolytes Research Center, Isfahan University of Medical Sciences, Isfahan, Iran; ^2^Department of Physiology, Isfahan University of Medical Sciences, Isfahan, Iran; ^3^Isfahan MN Institute of Basic & Applied Sciences Research, Isfahan, Iran; ^4^Department of Clinical Pathology, Isfahan University of Medical Sciences, Isfahan, Iran

**Keywords:** Renal ischemia reperfusion, S-methylisothiourea, Renal injury, Nitric oxide

## Abstract

**Introduction:** Excessive production of nitric oxide (NO) via inducible nitric oxide synthase (iNOS) is associated in renal ischemia reperfusion injury (IRI).

**Objectives:** This study was designed to investigate the role of S-methylisothiourea (SMT) as selective inhibitor iNOS in renal IRI. Materials and Methods: Male Wistar rats were subjected to 45 minutes of bilateral renal ischemia by occlusion of renal vessels of both kidney followed by 24 hours of reperfusion. Prior to renal IRI, the rats received either vehicle (saline, group 2) or SMT (50 mg/kg, group 3), and were compared with the sham-operated animals (group 1). At the end of reperfusion period, the rats were sacrificed for kidney tissue pathology investigation.

**Results:** Serum creatinine (Cr), blood urea nitrogen (BUN), nitrite levels, and kidney weight significantly increased in groups 2 and 3 (*P* < 0.05). Kidney tissue damage scores in groups 2 and 3 were also higher than that in the sham-operated group (*P * < 0.05).

**Conclusion:** SMT not only prevent the kidney during IRI, but also promotes kidney function disturbance and severity of renal injury.

**Implication for health policy/practice/research/medical education:**

In an experimental investigation on rats, we found, S-methylisothiourea not only prevent the kidney during ischemia/reperfusion injury, but also promotes kidney function disturbance and severity of renal injury.

## Introduction

Kidney ischemia is the most common disturbance in clinic, accompanied with renal failure (1). However, still there is no practical sufficient solution for amelioration of acute renal failure (ARF) as a consequence of ischemia reperfusion injury (IRI) (2,3). Usually, a decrease in renal blood for several minutes and then restoration of the blood flow may result in ARF (4,5). ARF is seen in different conditions such as gut ischemia (5), cardiopulmonary bypass (6), myocardial infarction (7) and stroke (8). Moreover, IRI plays a major role in short or long-term graft rejection in organ transplantations (5,9,10). It is reported that slight changes in total renal blood flow may lead to anoxic injury in the medulla, tubular dysfunction, salt wasting, and glomerular vasoconstriction (11,12). IRI also may disturb other organs such as circulatory (13) and pulmonary systems (14,15); therefore, it shows the complexity of the systemic response to kidney IRI (15). As IRI is a major cause of mortality, it is important to find a way for reducing harmful metabolites induced during IRI.

Nitric oxide (NO) is an important molecule both in physiological and pathophysiological conditions (16-18). NO is synthesized from L-arginine, and this free radical is produced from three isoforms of NO synthase (NOS). The inducible NOS (iNOS) can be produced in the kidney (19,20) by inducible factors; cytokines and lipopolysaccharide (21,22). It is reported that under IRI condition, the renal cell can induce iNOS (18). Although NO has important roles in the homeostatic regulation of glomerular, vascular, and tubular functions (23-27), the excessive amount of NO result in pathophysiological conditions especially in IRI, and excessive NO worsens renal injury during ischemia. Accordingly, iNOS inhibition may improve or prevent destructive effects of IRI (28-31). A study indicated that S-methylisothiourea (SMT), an iNOS inhibitor, plays differential roles in sepsis-associated multiple organ dysfunctions (32,33).

### Objectives

We hypothesized that SMT may protect the kidney against IRI. To confirm this hypothesis, SMT was administered 2 hour before ischemia then the kidney was perfused for 24 hours.

## Materials and methods

### Animals

Male Wistar rats weighting 160-220 g were housed at the room temperature of 23-25°C with a 12-hour light/dark cycle. The rats were fed with rat chow and water *ad libitum*. The experiment protocol was in advance approved by the Isfahan University of Medical Sciences Ethics Committee.

### Experimental protocol

The rats were randomly assigned to IRI (group 2, n = 7) and IR + SMT group (group 3, n = 5). At the first day of the experiment, the animals in these groups received a single dose of saline or SMT (50 mg/kg), respectively, 2 hours prior to ischemia. SMT was purchased from Sigma (St. Louis, Missouri, USA). To induce ischemia, all the rats were anesthetized with the mixture of xylazine (10 mg/kg, i.p) and ketamine (75 mg/kg, i.p). Incisions were made and the kidneys were excised with care. The renal artery and vein were occluded in both kidneys by placing a clamp around the vessels for 45 minutes. Then, the clamp was removed with care to make sure that blood flows into the kidneys. The same surgical procedure was done on the animals in group 1 except clamping the vessels. The animals were kept in the animal room and 24 hours later, they were anesthetized again to obtain blood sample by heart puncture and then sacrificed. The kidneys were removed rapidly for histology procedures and measurement. The left kidney was fixed in 10% formalin solution, embedded in paraffin for histopathological staining. The hematoxylin and eosin staining was applied to examine the tissue injury. To consider the kidney damage, the pathologist evaluated presence of tubular atrophy, hyaline casts, ischemic necrosis, vacuolization, and debris. The damages were scored from 1-4, where 0 was assigned to normal tissue. The right kidney was homogenized, and centrifuged at 6000 g for 10 minutes. The supernatant was removed and the sample was centrifuged again at 15000 g for 2 minutes for measuring selected biochemical parameters.

### Measurements

Serum creatinine (Cr) and blood urea nitrogen (BUN) levels were determined using quantitative kits (Pars Azmoon, Iran) and autoanalyzer (Technicon, RA1000). Levels of nitrite (stable NO metabolite) in the serum and kidney were measured using a colorimetric assay kit. The serum level of malondialdehyde (MDA) was quantified according to the manual method.

### Statistical analysis

The data are presented as mean ± standard error of the mean. The groups were compared with each other by one-way analysis of variance (ANOVA), followed by the least significant difference (LSD) with regard to the serum levels of BUN, Cr, nitrite, and MDA; and kidney tissue levels of MDA and nitrite, kidney weight (KW), and bodyweight (BW) changes. The Mann-Whitney or Kruskal-Wallis tests were used to compare the pathological damage score of the groups. *P<*0.05 was considered statistically significant.

## Results

### Effect of IR or IR + SMT on serum BUN and Cr levels

**Figure 1:**
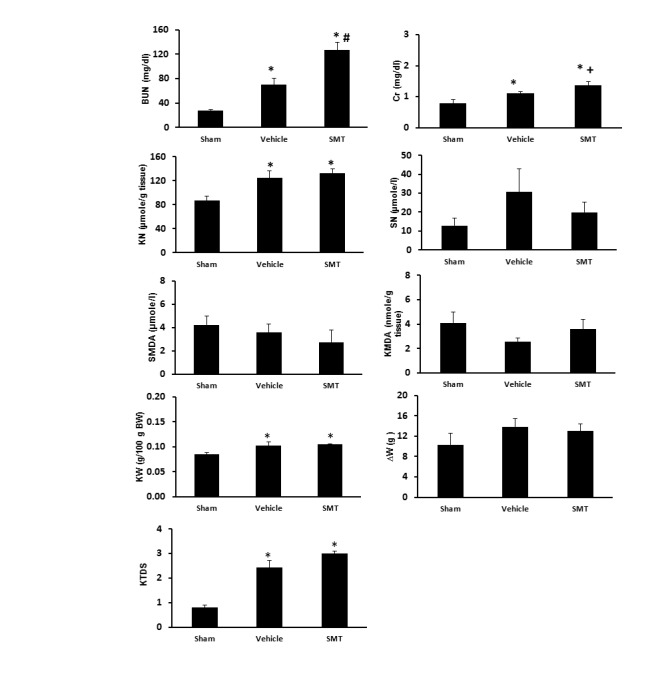
The serum levels of Cr and BUN in three experimental groups. *indicates significant difference from the sham-operated group (*P*<0.05); # and + show significant difference from IR + saline group at the level of *P*<0.05 and *P*<0.1, respectively.

The serum levels of BUN and Cr significantly increased in the control group (IR + saline) (*P<*0.05). However, administration of SMT did not decrease the serum levels of Cr and BUN toward normal. The serum levels of BUN and Cr in IR + SMT group (group 3) was significantly higher than those in the control group (*P<*0.05). This data did not show any protective role of SMT against kidney IRI (Figure 1).

### Effect of IR or IR + SMT on kidney weight and damage

The KW significantly increased in IR + saline and IR + SMT groups when compared with the sham-operated group (*P<*0.05). The results of renal histopathology demonstrated significant increase of tissue damage in IR + saline and IR + SMT groups compared with the sham-operated group (*P<*0.05). In addition, this result shows that administration of SMT can lead to further damage in kidney in addition to the renal IRI (Figure 1).

### Effect of IR or IR + SMT on serum MDA and nitrite levels

**Figure 2:**
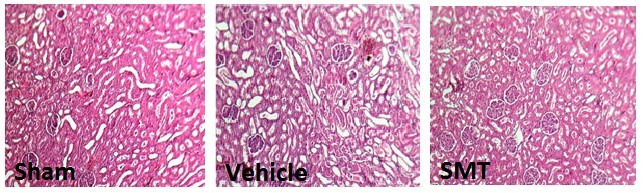
Sample images of kidney tissue in three experimental groups. Higher tissue damage was observed in the IR + SMT group.

The nitrite levels in the serum and kidney tissue of IR + saline and IR + SMT groups were higher than those in the sham-operated group. However, this increase was statistically significant for kidney tissue (*P<*0.05). The groups were not significantly different in terms of serum and kidney tissue levels of MDA (Figure 2).

## Discussion

In the current study, we investigated the effect of SMT, a potent and selective iNOS inhibitor, on renal IRI. Renal IRI induced ARF in animal model, which is characterized by increase in serum BUN and Cr levels, KW, and KTDS. Other findings were in agreement with these observations (34,35). During IRI reactive oxygen species are produced and alter tubular permeability, which result in tubular damage, glomerular injury, and renal dysfunction (36-38). Shoskes et al showed 60 minutes of ischemia followed by 2 hours of reperfusion increased NOS activity (39). Some evidence has shown excessive NO production during IRI is related to renal dysfunction (39,40). Administration of SMT promoted renal damage induced by IRI. Contrary to our findings, Guven et al showed that administration of SMT 6 hours prior to renal ischemia followed by 6 hours of reperfusion ameliorated renal dysfunction (33). This contrast may be related to the different protocols used. Although reperfusion is necessary for survival in ischemic kidney, it leads to further injury in the tissue (41,42) probably due to distribution of harmful metabolites (4,43). In the present study, renal reperfusion was achieved for 24 hours that may lead to further injury.

Administration of SMT increased lipid peroxidation and hepatic injury, however, it seems that NO has cytoprotective and cytotoxic roles. For example, L-Arg as the NO donor ameliorates cisplatin-induced nephrotoxicity in male rats (44,45), and S-Nitroso-N-acetylpenicillamine (SNAP) as another NO donor abolished hepatic injury (45) while L-NAME as the NO inhibitor accelerated nephrotoxicity induced by cisplatin (46). Inhibition of NO by SMT reduced renal dysfunction induced by IRI (33). Hsu et al reported the protective effect of iNOS in hepatic IRI, which was proved via NO donor effect on increment of iNOS activity and consequent decrement of MDA level (45). It seems that in our study, SMT inhibited beneficial effect of iNOS. On the other hand, in this study, despite inhibition of iNOS, SMT did not decrease kidney nitrite level. It is reported that iNOS and eNOS participate in production of nitrite during IRI (40). Therefore, increased kidney NO level in SMT-treated group possibly originated from eNOS.

Our findings showed that SMT reduced the enhanced serum NO induced by IRI, although it was not significant. Other investigations demonstrated decrement in plasma nitrite/nitrate levels by iNOs inhibitors (30). They indicated that iNOS inhibitors reduce peroxynitrite formation possibly due to inhibition of iNOS activity, which in turn led to decreased NO levels. Finally, the increased KW by IRI is probably related to edema and renal cell proliferation (40,47) and SMT did not affect it.

## Conclusion

ARF due to IRI is a complex disorder which is involved many physiology and pathology pathways including NO system that need to be determined. NOS inhibition by SMT during IRI increased kidney injury possibly due to disturbance of renal blood flow and oxidative stress.

## Authors’ contribution

All authors contributed to manuscript equally.

## Conflicts of interest

The authors declared no competing interests.

## Ethical considerations

Ethical issues (including plagiarism, data fabrication, double publication) have been completely observed by the authors.

## Funding/Support

This research was supported by Isfahan University of Medical Sciences.

## References

[1] Star RA (1998). Treatment of acute renal failure. Kidney Int.

[2] Deng J, Hu X, Yuen PS, Star RA (2004). Alpha-melanocyte-stimulating hormone inhibits lung injury after renal ischemia/reperfusion. Am J Respir Crit Care Med.

[3] Rabb H, Bonventre JV. Experimental strategies for acute renal failure-the future. In: Therapy in Nephrology and Hypertension. Philadelphia: WB Saunders; 1999:72-80.

[4] Liano F, Pascual J (1996). Epidemiology of acute renal failure: a prospective, multicenter, community-based study Madrid Acute Renal Failure Study Group. Kidney Int.

[5] Hearse DJ, Bolli R (1992). Reperfusion induced injury: manifestations, mechanisms, and clinical relevance. Cardiovasc Res.

[6] Kirklin JK, Westaby S, Blackstone EH, Kirklin JW, Chenoweth DE, Pacifico AD (1983). Complement and the damaging effects of cardiopulmonary bypass. J Thorac Cardiovasc Surg.

[7] Anavekar NS, McMurray JJ, Velazquez EJ, Solomon SD, Kober L, Rouleau JL (2004). Relation between renal dysfunction and cardiovascular outcomes after myocardial infarction. N Engl J Med.

[8] Khosla S, Kunjummen B, Manda R, Khaleel R, Kular R, Gladson M (2003). Prevalence of renal artery stenosis requiring revascularization in patients initially referred for coronary angiography. Catheter Cardiovasc Interv.

[9] Pagtalunan ME, Olson JL, Tilney NL, Meyer TW (1999). Late consequences of acute ischemic injury to a solitary kidney. J Am Soc Nephrol.

[10] Prokai A, Fekete A, Banki NF, Muller V, Ver A, Degrell P (2011). Renoprotective effect of erythropoietin in rats subjected to ischemia/reperfusion injury: gender differences. Surgery.

[11] Brezis M, Rosen S (1995). Hypoxia of the renal medulla--its implications for disease. N Engl J Med.

[12] Wang Z, Rabb H, Craig T, Burnham C, Shull GE, Soleimani M (1997). Ischemic-reperfusion injury in the kidney: overexpression of colonic H+-K+-ATPase and suppression of NHE-3. Kidney Int.

[13] Kelly KJ (2003). Distant effects of experimental renal ischemia/reperfusion injury. J Am Soc Nephrol.

[14] Rabb H, Chamoun F, Hotchkiss J (2001). Molecular mechanisms underlying combined kidney-lung dysfunction during acute renal failur. Contrib Nephrol.

[15] White LE, Hassoun HT (2012). Inflammatory mechanisms of organ crosstalk during ischemic acute kidney injury. Int J Nephrol.

[16] Bredt DS, Snyder SH (1994). Nitric oxide: a physiologic messenger molecule. Annu Rev Biochem.

[17] Gross SS, Wolin MS (1995). Nitric oxide: pathophysiological mechanisms. Annu Rev Physiol.

[18] Kone BC (1997). Nitric oxide in renal health and disease. Am J Kidney Dis.

[19] Liang M, Knox FG (2000). Production and functional roles of nitric oxide in the proximal tubule. Am J Physiol Regul Integr Comp Physiol.

[20] Shultz PJ, Tayeh MA, Marletta MA, Raij L (1991). Synthesis and action of nitric oxide in rat glomerular mesangial cells. Am J Physiol.

[21] Chatterjee PK, Hawksworth GM, McLay JS (1999). Cytokine-stimulated nitric oxide production in the human renal proximal tubule and its modulation by natriuretic peptides: A novel immunomodulatory mechanism?. Exp Nephrol.

[22] Alexander C, Rietschel ET (2001). Bacterial lipopolysaccharides and innate immunity. J Endotoxin Res.

[23] Kone BC, Baylis C (1997). Biosynthesis and homeostatic roles of nitric oxide in the normal kidney. Am J Physiol.

[24] Navar L, Inscho E, Majid S, Imig J, Harrison-Bernard L, Mitchell K (1996). Paracrine regulation of the renal microcirculation. Physiol Rev.

[25] Raij L, Baylis C (1995). Glomerular actions of nitric oxide. Kidney Int.

[26] Thorup C, Persson AE (1994). Inhibition of locally produced nitric oxide resets tubuloglomerular feedback mechanism. Am J Physiol.

[27] Wilcox CS, Welch WJ, Murad F, Gross SS, Taylor G, Levi R (1992). Nitric oxide synthase in macula densa regulates glomerular capillary pressure. Proceedings of the National Academy of Sciences.

[28] Chatterjee PK, Patel NS, Sivarajah A, Kvale EO, Dugo L, Cuzzocrea S (2003). GW274150, a potent and highly selective inhibitor of iNOS, reduces experimental renal ischemia/reperfusion injury. Kidney Int.

[29] Mark LA, Robinson AV, Schulak JA (2005). Inhibition of nitric oxide synthase reduces renal ischemia/reperfusion injury. J Surg Res.

[30] Chatterjee PK, Patel NS, Kvale EO, Cuzzocrea S, Brown PA, Stewart KN (2002). Inhibition of inducible nitric oxide synthase reduces renal ischemia/reperfusion injury. Kidney Int.

[31] Qi S, Xu D, Ma A, Zhang X, Chida N, Sudo Y (2006). Effect of a novel inducible nitric oxide synthase inhibitor, FR260330, in prevention of renal ischemia/reperfusion injury in vervet monkeys. Transplantation.

[32] Mitaka C, Hirata Y, Masaki Y, Takei T, Yokoyama K, Imai T (2000). S-Methylisothiourea sulfate improves renal, but not hepatic dysfunction in canine endotoxic shock model. Intensive Care Med.

[33] Guven A, Uysal B, Akgul O, Cermik H, Gundogdu G, Surer I (2008). Scavenging of peroxynitrite reduces renal ischemia/reperfusion injury. Ren Fail.

[34] Azarkish F, Nematbakhsh M, Fazilati M, Talebi A, Pilehvarian AA, Pezeshki Z (2013). N-acetylcysteine Prevents Kidney and Lung Disturbances in Renal Ischemia/Reperfusion Injury in Rat. Int J Prev Med.

[35] Moeini M, Nematbakhsh M, Fazilati M, Talebi A, Pilehvarian AA, Azarkish F (2013). Protective role of recombinant human erythropoietin in kidney and lung injury following renal bilateral ischemia-reperfusion in rat model. Int J Prev Med.

[36] Paller MS, Hoidal JR, Ferris TF (1984). Oxygen free radicals in ischemic acute renal failure in the rat. J Clin Invest.

[37] Bonventre JV (1993). Mechanisms of ischemic acute renal failure. Kidney Int.

[38] Baker GL, Corry RJ, Autor AP (1985). Oxygen free radical induced damage in kidneys subjected to warm ischemia and reperfusion Protective effect of superoxide dismutase. Ann Surg.

[39] Shoskes DA, Xie Y, Gonzalez-Cadavid NF (1997). Nitric oxide synthase activity in renal ischemia-reperfusion injury in the rat: implications for renal transplantation. Transplantation.

[40] Vinas JL, Sola A, Genesca M, Alfaro V, Pi F, Hotter G (2006). NO and NOS isoforms in the development of apoptosis in renal ischemia/reperfusion. Free Radic Biol Med.

[41] Greene EL, Paller MS (1991). Oxygen free radicals in acute renal failure. Miner Electrolyte Metab.

[42] Weinberg JM (1991). The cell biology of ischemic renal injury. Kidney Int.

[43] Mehta JL, Nichols WW, Mehta P (1988). Neutrophils as potential participants in acute myocardial ischemia: relevance to reperfusion. J Am Coll Cardiol.

[44] Eshraghi-Jazi F, Nematbakhsh M, Nasri H, Talebi A, Haghighi M, Pezeshki Z (2011). The protective role of endogenous nitric oxide donor (L-arginine) in cisplatin-induced nephrotoxicity: gender related differences in rat model. J Res Med Sci.

[45] Hsu CM, Wang JS, Liu CH, Chen LW (2002). Kupffer cells protect liver from ischemia-reperfusion injury by an inducible nitric oxide synthase-dependent mechanism. Shock.

[46] Moslemi F, Nematbakhsh M, Eshraghi-Jazi F, Talebi A, Nasri H, Ashrafi F (2013). Inhibition of nitric oxide synthase by L-NAME promotes cisplatin-induced nephrotoxicity in male rats. ISRN Toxicol.

[47] Forbes JM, Hewitson TD, Becker GJ, Jones CL (2000). Ischemic acute renal failure: long-term histology of cell and matrix changes in the rat. Kidney Int.

